# Nanolayered Metal Phosphates as Biocompatible Reservoirs for Antimicrobial Silver Nanoparticles

**DOI:** 10.3390/ma14061481

**Published:** 2021-03-18

**Authors:** Inés García, Camino Trobajo, Zakariae Amghouz, Alaa Adawy

**Affiliations:** 1 Nanomaterials and Nanotechnology Research Centre—CINN (CSIC), 33940 El Entrego, Spain; garciagonzalezines@hotmail.com (I.G.); ctf@uniovi.es (C.T.); 2 Department of Organic and Inorganic Chemistry, University of Oviedo, 33006 Oviedo, Spain; 3 Department of Material Science and Metallurgical Engineering, University of Oviedo, 33203 Gijón, Spain; amghouzzakariae@uniovi.es; 4 Laboratory of High-Resolution Transmission Electron Microscopy, Institute for Scientific and Technological, Resources, University of Oviedo, 33006 Oviedo, Spain

**Keywords:** inorganic layered nanomaterials, zirconium phosphate, titanium phosphate, silver nanoparticles, cytocompatibility, antimicrobial activity, bone cement

## Abstract

There is an increasing demand on synthesizing pharmaceuticals and biomaterials that possess antimicrobial and/or antiviral activities. In this respective silver nanoparticles are known for their excellent antimicrobial activity. Nevertheless, their uncontrolled release in a biological medium can induce a cytotoxic effect. For this, we explored the use of nanolayered metal phosphates based on titanium and zirconium as materials that can be enriched with silver nanoparticles. Employing the hydrothermal route, crystalline α-phases of zirconium and titanium phosphates (α-ZrP, α-TiP) were synthesized and there after surface-enriched with silver nanoparticles. The structural assessment confirmed the stability of the structures and their sizes are in the nanoscale at least in one dimension. The cytocompatibility assays confirmed the biocompatibility of the pristine phases and the antimicrobial assay confirmed that both silver-enriched nanolayered structures maintain an antibacterial effect at reasonably low concentrations.

## 1. Introduction

Following the golden era of antibiotics, the increasing awareness of the danger we face in the current era of antimicrobial resistance, has directed the researchers to investigate the possibility of synthesizing new combination of materials that could accomplish the required antimicrobial effects [1]. Among the materials that could provide an alternative route and promising results are the metal phosphates [2,3].

Zirconium phosphate has been under investigation since 1956 when its cation exchange properties were first reported [4]. Since then, it was known as an amorphous phase until its first crystalline structure was reported in 1964 as Zr(HPO_4_)_2_·H_2_O (abbreviated as α-ZrP) [5]. Many studies were performed to develop new synthesis methods for ZrP-materials and to explore its prospective applications, because of its outstanding chemical and physical properties [6,7,8]. Among these methods, the hydrothermal method has the advantage of resulting in highly crystalline layered α-ZrP structures with a narrow particle size distribution and controlled morphology [9]. α-ZrP has gained a considerable interest owing to the ease of controlling its structure and facile exfoliation into few or single-layered entities with all the consequent possibilities of enhancing its intercalation capacity and increasing tremendously its surface area to mass ratio [10,11,12,13,14,15,16].

On the other hand, the investigations on titanium phosphate, α-TiP, as another crystalline tetravalent metal phosphate started about a decade later as a consequence of the success reported on its zirconium counterpart [17,18]. The main characteristic that was reported is the high capacity of α-TiP to undergo ion exchange [19]. In addition, many studies focused on its functionalization through the intercalation with amines and its exfoliation that led to several potential applications [20,21,22,23].

Questioning the suitability of these inorganic layered nanomaterials for biomedical applications, several recent studies have confirmed the biocompatibility of α-ZrP and this facilitated the route towards considering it for this purpose [24,25,26]. Beside its biocompatibility, the physical properties of α-ZrP, in particular: crystalline nature, adjustable particle size, high ion-exchange capacity and above all its non-spherical shape, being inorganic layered nanomaterial, made α-ZrP a suitable choice for investigating its capabilities in applications related to drug and gene delivery [27,28]. On the contrary, to our knowledge no investigations were performed to study the biocompatibility of the α-TiP despite the possibility that it could share α-ZrP some of the above-mentioned biologically-relevant physical and chemical properties beside its recently confirmed stability in aqueous media [29].

In fact, the most relevant aspects for considering these inorganic nanolayered structures for biomaterials’ applications are their ability to provide controlled release of a loaded drug, nanoparticles, or ionic species in addition to their non-spherical morphology which improves their margination and adherence to biological tissue [30,31,32]. These characteristics could make α-ZrP and α-TiP interesting alternatives for bone cement applications especially if they are enriched with functional nanoparticles [33,34,35]. Silver is one of the most widely known elements regarding their biofunctional capabilities, including wide ranging antimicrobial effects against Gram-positive and Gram-negative bacteria, fungi, protozoa and certain viruses including antibiotic-resistant strains [36]. Furthermore, silver is reported for its ability to reduce infections in the treatment of burned areas [37]. When silver is prepared as nanoparticles, it provides excellent antimicrobial effects [38,39].

Therefore, in the present study we used the hydrothermal methodology to synthesize nanolayered crystalline α-ZrP and α-TiP with narrow size distribution. Using a thermal treatment, these phases were loaded homogenously with crystalline silver nanoparticles possessing spherical morphology and averaged around 5 nm with very narrow size distribution to ensure their bio-effectiveness. Beside the structural assessment, we studied the antimicrobial activity and cytocompatibility of the resultant nanolayers. The results support our hypothesis of the potential benefits of using these nanolayered metal phosphates and particularly introduce the α-TiP as a strong competing alternative to α-ZrP for biomedical applications.

## 2. Materials and Methods

### 2.1. Preparation of α-TiP Nanolayered Crystals & Their Enrichment with Silver Nanoparticles (AgNPs)

The preparation procedure consisted of two stages; synthesis of an amorphous phase of titanium phosphate [15], followed by the synthesis of the crystalline α-titanium phosphate. For this, a total mass of 25 g of TiCl_4_ was dissolved in 430 mL of 2 M HCl. The resultant solution was slowly dropwise added to a beaker containing 400 mL of 1.25 M H_3_PO_4_ under stirring. The resultant mixture was left to rest for 24 h. A thick white gel was obtained which was centrifuged at 1489× *g*, filtered from the supernatant solution and washed with 3% H_3_PO_4_ to remove chlorides and afterwards was air-dried at room temperature. This procedure resulted in the formation of an amorphous phase of titanium phosphate, from which 1.5 g were mixed with 30 mL of 5 M H_3_PO_4_ in Teflon-lined stainless-steel hydrothermal autoclave reactor (40 mL) that was heated at 180 °C for 48 h. At the end of the hydrothermal reaction the obtained mixture has become very acidic (pH = 0.4) and layered crystals were washed thoroughly with water, filtered and air-dried. To enrich the nanocrystals with silver nanoparticles, a dry mixture of 0.7007 g of α-TiP and 0.9223 g of AgNO_3_ (the stoichiometric amounts for a full H^+^/Ag^+^ ion-exchange process on α-TiP) was prepared and heat-treated at 230 °C for 6 days. The resultant Ag-enriched α-TiP nanocrystals were washed, filtered and air-dried at room temperature.

### 2.2. Preparation of α-ZrP Nanolayered Crystals & Their Enrichment with AgNPs

Likewise, the procedure consisted of two stages and the first of which relied on a methodology reported elsewhere [16]. A mass of 25 g of ZrCl_4_ was dissolved in 430 mL of 2 M HCl. The resultant solution was then added through titration to a beaker containing 400 mL of 1.25 M H_3_PO_4_ under stirring. The resultant mixture was left sealed for 24 h. A thick white gel was obtained which was filtered from the supernatant solution and washed with 3% H_3_PO_4_ to remove chlorides and afterwards was air-dried at room temperature. This procedure resulted in the formation of an amorphous phase of zirconium phosphate, from which 4.5 g were mixed with 20 mL of 15 M H_3_PO_4_ in Teflon-lined stainless-vessel hydrothermal autoclave reactor (40 mL) that was incubated for 6 days at 200 °C. At the end of the hydrothermal reaction the obtained mixture has become acidic (pH = 2.01) and the resultant nanolayered crystals were washed thoroughly with water filtered and air-dried. To enrich the nanolayered crystals with silver nanoparticles, a dry mixture of 0.7001 g of α-ZrP and 0.7900 g of AgNO_3_ (stoichiometric amounts for a full H^+^/Ag^+^ ion-exchange process on α-ZrP) was prepared and heat-treated for 6 days at 230 °C. The resultant Ag-enriched α-ZrP nanocrystals were washed, filtered and air-dried at room temperature.

### 2.3. Structural Characterization Methodologies

The powder X-ray diffraction (PXRD) patterns were recorded on an X’Pert diffractometer (Philips, Amelo, The Netherlands) with Cu-Kα radiation (λ = 1.5406 Å) at room temperature over the angular 2θ range 5–45° with a step of 0.02° and a counting time of 0.4 s/step. Top-view scanning electron microscopy (SEM) micrographs for secondary and back-scattered electrons (SE and BSE) and energy dispersive X-ray microanalysis (EDX) were recorded with a JEOL JSM-6100 scanning electron microscope (JEOL, Tokyo, Japan) operating at 20 kV coupled with an X-Max silicon drift detector (SDD) 80 mm^2^ energy dispersive X-ray spectroscopy (EDS) detector (Oxford instruments, High Wycombe, England). The (High-Resolution) Transmission Electron Microscopy ((HR)TEM) studies were performed on a JEOL JEM-2100F transmission electron microscope (JEOL, Tokyo, Japan) operating at an accelerating voltage of 200 kV and equipped with a field-emission gun and an ultra-high-resolution pole-piece that provided a point-resolution better than 1.9 Å. This TEM is also equipped with scanning transmission electron microscope (STEM) control unit (Gatan), energy dispersive X-ray spectroscopy (EDS) detector (Oxford Instruments, X-Max (SDD) 80 mm^2^, High Wycombe, England), CCD camera (Gatan 14-bit Orius SC600, GATAN, Pleasanton, CA, USA), and bright-field (BF) and high-angle annular dark field (HAADF) detectors (JEOL). This microscope was used to perform TEM, HRTEM, selected area electron diffraction (SAED), STEM (BF and HAADF) and EDX-STEM (line-scan and area mapping) analysis. Fine powder of every sample was dispersed in ethanol, shortly sonicated and sprayed on a Lacey-carbon-on-copper grid (200 mesh, EM science, Hatfield, UK), and then allowed to air dry. Afterwards, the dried grid was mounted on a JEOL single-tilt holder. Acquiring, processing and analysis of all micrographs were executed using the suite of Gatan Digital Micrograph software (version 2.32.888.0). Quantitative analyses were done using INCA Microanalysis Suite software (version 4.15).

### 2.4. Studying the Antimicrobial Activity of α-ZrP and α-TiP Phases before and after AgNPs Incorporation

Two parameters were under test: Minimum Inhibitory Concentration (MIC) and Minimum Bactericidal Concentration (MBC). MIC of a certain agent is the minimum concentration of the agent at which no microorganism development is observed. MBC is the minimum concentration that can kill a bacterial strain (death of 99.9% of the inoculum). The protocol ASTM E-2149-01 (Antimicrobial Activity of Immobilized Agents Under Dynamic Contact Conditions) describes a test method that allows the measurement of the antimicrobial activity of a material or product under dynamic contact conditions. The purpose of this test is to examine the antimicrobial activity of a material against a suspension of a particular microorganism. This is achieved by exposing the microorganism to the material to be tested under dynamic conditions (agitation) and analyzing the number of surviving microorganisms after certain times. In this case, the microorganism is exposed to a series of serial dilutions of the agent. Before starting the experiment, the material to be tested (α-ZrP and α-TiP) was sterilized in an autoclave at 121 °C for 20 min. The strain used as inoculum was *Escherichia coli* ATCC 8739 at an initial concentration of 1.2 × 10^6^ CFU mL^−1^ (CFU: colony forming unit). To determine the MIC, liquid Nutrient Broth (NB) was used as culture media in which concentrations of 0.0125, 0.025, 0.05, 0.075 and 0.1 mg·mL^−1^ of α-TiP and α-ZrP phases were introduced. The experiments were run in triplicates. The tubes were set in an incubator overnight (≈24 h) at 37 °C. After the incubation period, the tubes that showed turbidity were trashed as this indicates bacterial growth. The clear tubes with lowest product concentration indicated the MIC. The solutions in all clear tubes were then used to prepare serial dilutions (10^−1^ down to 10^−8^) of every concentration, this time, in Petri dishes containing solid NB: 1.5% agar. Afterwards, the prepared Petri dishes were incubated for other 24 h at 37 °C. A decrease in the number of counted colonies proportional to the dilution factor is a good indication of the success of the experiment. The Petri dishes with no observed microbial colonies (completely clear) indicates the concentration of the tested material corresponding to the MBC.

### 2.5. Studying Cell Proliferation in the Presence of α-ZrP and α-TiP Phases before and after Their Enrichment with AgNPs

Cell line Saos-2 (Sarcoma osteogenic) was used in this assay. The cells were cultured in a complete Dulbecco′s Modified Eagle′s–Medium (DMEM) containing 10% Fetal Bovine Serum (FBS) and 1% penicillin/streptomycin at 37 °C in humidified incubator containing 5% CO_2_. Cell Counting Kit 8 (CCK-8; Sigma, St. Louis, MO, USA) was used to determine the cell viability of Saos2. The cells were seeded at 2 × 10^3^/well into 96-well plate and cultured for 24 h in DMEM-10% FBS growth medium. The experiments were designed in triplicates to confirm their reproducibility. Equal masses of the phases’ powders were then placed in contact with the cells and serial dilutions were prepared. The microplate was then incubated for 7 days. The powders were filtered out and 100 µL of DMEM-10%FBS and 10 µL CCK-8 reagent were added to each well and incubated for additional 3 h. The absorbance of each well was determined at 450 nm in an Elx800 plate reader (Biotek, Winooski, VT, USA). The obtained absorbance value is proportional to the survival of the cells. The cell viability rate was determined by calculating the percentage ratio of the absorbance at 450 nm from a well of a given powder concentration to the absorbance from a control well with cells, but no powder.

## 3. Results

**Figure 1:**
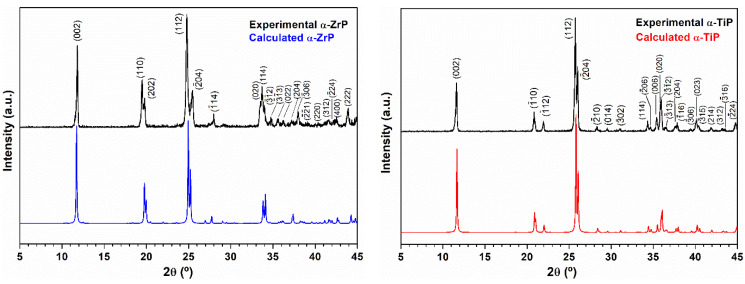
**Left panel**: Experimental X-ray diffraction pattern (black) of the obtained zirconium phosphate powder using the hydrothermal treatment. For comparison, the pattern displayed in blue is for the calculated PXRD pattern of the layered α-Zr(HPO_4_)_2_.H_2_O phase (ICSD 3 10258: Monoclinic, P2_1_/c, a = 9.0760 Å, b = 5.2980 Å, c = 16.2200 Å and β = 111.5000° [15]; **Right panel**: Experimental X-ray diffraction pattern (black) of the obtained titanium phosphate powder using the hydrothermal treatment. For comparison the pattern displayed in red is for the calculated PXRD pattern of the layered α-Ti(HPO_4_)_2_·H_2_O phase (ICSD PDF4 01-086-1364: Monoclinic, P2_1_/c, a = 8.6110 Å, b = 4.9933 Å, c = 16.1507 Å and β = 110.206° [18].

Powder XRD confirmed the successful synthesis of crystalline zirconium phosphate and titanium phosphate in their α-phases (Figure 1), although for the latter, we applied a different preparation methodology than the mostly reported one to result in nanolayered crystalline structures with narrow size distribution.

**Figure 2:**
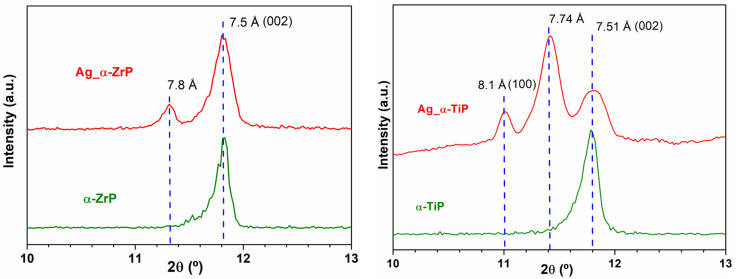
A magnified view for the peaks appeared in the range of 2θ = 10–13° in PXRD that corresponds to the inter layer distances of α-ZrP and α-TiP [21]. **Left panel**: Experimental X-ray diffraction pattern (red) of the obtained Ag-enriched zirconium phosphate compared to the experimental X-ray diffraction pattern of the pristine α-ZrP (green); **Right panel**: Experimental X-ray diffraction pattern (red) of the obtained Ag-enriched titanium phosphate compared to the experimental X-ray diffraction pattern of the pristine α-TiP (green).

After the Ag-enrichment treatment, the powder X-ray diffraction patterns for α-ZrP showed that the first peak appearing at 2θ ≈ 11.82° in pristine α-ZrP has become preceded with one more peak at 2θ ≈ 11.32° in Ag-α-ZrP. Whereas, for Ag-α-TiP this first peak appearing at 2θ ≈ 11.78° in pristine α-TiP, has become preceded with two additional peaks at 2θ ≈ 11.02° and 11.42° (Figure 2). In the pristine phases, the peaks observed at 2θ ≈ 11.82° (α-ZrP) and 2θ ≈ 11.78° (α-TiP) in the PXRD patterns of both crystalline structures correspond to the d*_002_*-spacings of the interlayer distances [23].

**Figure 3:**
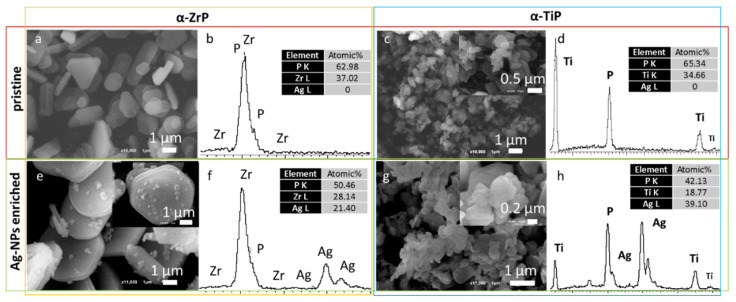
SEM micrographs for the pristine (**upper row**) and Ag-enriched (**lower row**) α-ZrP (**left panel**) and α-TiP (**right panel**) nanolayered crystals and their corresponding EDX spectral patterns along with their normalized atomic percentages of silver, phosphorus and zirconium or titanium (beside each micrograph).

The SEM imaging revealed that the adopted preparation methodology resulted in the formation of very thin hexagonally shaped crystals of the both zirconium and titanium phosphate phases (Figure 3, upper row). The α-ZrP crystals are bigger with crystals reach beyond 1 µm in their longest dimension, whereas the α-TiP crystals seldomly reached the 1 µm length. The EDX spectral analysis showed that the atomic ratio of the zirconium (or titanium) to phosphorus stayed around 1:2, which is consistent with what is expected from their respective chemical formula (Zr(HPO_4_)_2_·H_2_O and Ti(HPO_4_)_2_·H_2_O). Although the Ag-enrichment procedure was similar for both phases, the EDX spectral analyses revealed that α-TiP gained on average double the silver content that was gained by their zirconium counterparts (Figure 3, lower row). In both cases, the morphology and EDX analyses of the nanolayered crystals are conserved after the Ag-enrichment treatment.

**Figure 4:**
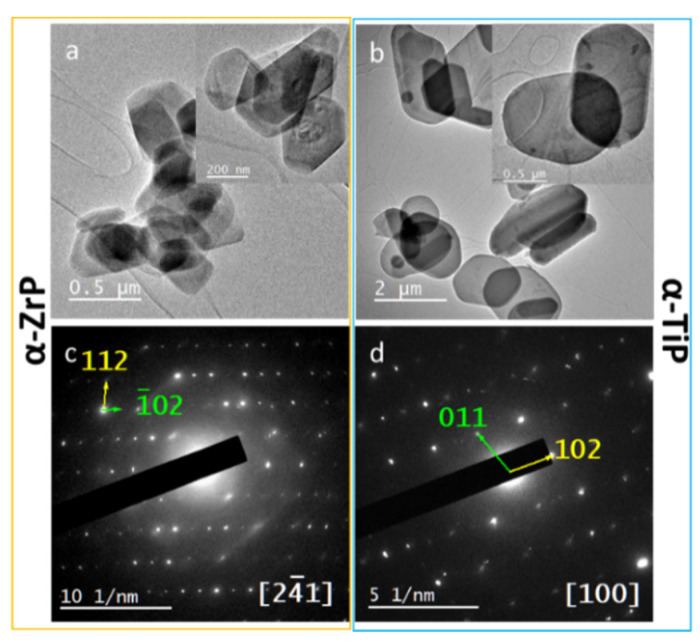
**Upper row**: TEM micrographs for the pristine α-ZrP (**a**) and α-TiP nanolayered crystals (**b**), showing their hexagonal morphology and minimal thickness; **Lower row**: SAED patterns for α-ZrP (**c**) and α-TiP (**d**) along zone axes (2$\bar{4}$1) and (100), respectively.

TEM provided a more detailed description and structural analysis for the four types of nanolayered crystals. TEM imaging of the pristine α-phases confirmed their morphology and minimal sizes determined for the nanolayered crystals ≈200 nm (Figure 4a,b) the crystals appear as nanoplates (thickness of 10–30 nm). In addition, SAED confirmed the crystalline nature of both phases and the analysis of the patterns resulted in confirming their structures which coincided with their PXRD data analysis (Figure 4c,d).

**Figure 5:**
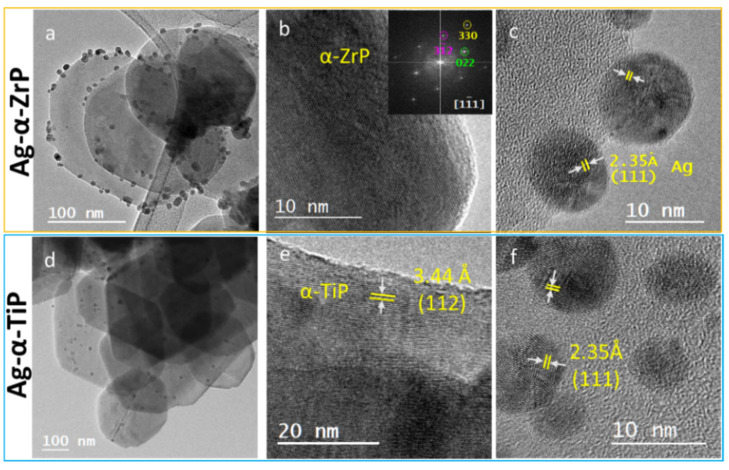
HRTEM micrographs for the Ag-enriched α-ZrP (**upper row**) and α-TiP nanocrystals (**lower row**) at increasing magnifications confirming the morphology as hexagonal shaped thin crystals (**a**,**d**); and crystalline structure with lattice fringes of d-spacings corresponding to the pristine structures of α-ZrP and α-TiP) (**b**,**e**); and their enrichment with crystalline silver nanoparticles with diameters ≤12 nm (**c**,**f**). The inset in (**b**) is the Fast Fourier Transform (FFT) of the HRTEM displayed micrograph.

Performing HRTEM imaging for the Ag-enriched nanolayered crystals provided a visible evidence for the preservation of the hexagonal crystal’s morphology and crystallinity, through the observation of lattice fringes on the crystals’ surfaces. In addition, silver is homogenously distributed on the surfaces of the α-ZrP and α-TiP nanolayered crystals, as nanoparticles possessing a spherical morphology. Measuring the observed d*_hkl_*-spacings on the crystals and nanoparticles confirmed their structural identity with the most prominent lattice fringes observed for AgNPs corresponds to (111) (Figure 5).

**Figure 6:**
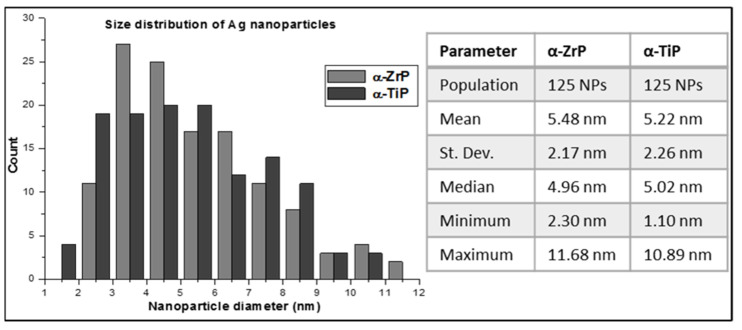
A graphical representation for size distribution of the AgNPs deposited on the surface of α-ZrP (light grey) and α-TiP (dark grey) nanocrystals. Statistics of the data are tabulated on the right side of the graph.

In order to perform a statistical analysis on the particle size distribution of the AgNPs on the α-phases of ZrP and TiP, multiple HRTEM images for the respective nanolayered crystals were collected and for each group the diameter of 125 AgNPs was precisely measured (Figure 6). For the α-ZrP, the AgNPs showed a perfect Gaussian distribution and had a slightly larger average size, compared with those loaded on the α-TiP, with the largest population of AgNPs possessing sizes in the range of 3–4 nm. On the other hand, AgNPs covering the α-TiP nanocrystals did not show a specific population with a preferable size and showed almost equal preferences for the size range 2–6 nm, with some nanoparticles as small as 1 nm. On both metal phosphate crystals, relatively low number of AgNPs had sizes greater than 9 nm.

For the purpose of studying the biofunctionality and biocompatibility of the synthesized nanolayered crystals, we explored two parameters: the antimicrobial activity and the cell viability rate. For the antimicrobial activity, at the tested concentrations (12.5, 25, 50, 75 and 100 µg·mL^−1^) we could not find any antimicrobial activity for the two pristine metal phosphate nanolayered crystals. On the other hand, the Ag-enriched α-ZrP and α TiP phases showed an antimicrobial activity at a lower concentration for the former compared to the latter (Table 1). For the two Ag-enriched metal phosphates the determined minimum inhibitory concentration (MIC) was found to be the same as their minimum bactericidal concentration (MBC).

To examine the cytocompatibility, loose powders of the respective pristine and Ag-enriched phases were used for the cell proliferation assay. Different concentrations for every phase were prepared relying on serial dilutions. As shown in Table 2, it appears that pristine α-ZrP and α-TiP are less cytotoxic when compared with their Ag-enriched phases. In all cases, concentrations around 125 mg·mL^−1^ were cytotoxic. Surprisingly, the cytocompatibility of α-TiP appears to be better than α-ZrP, the same for their Ag-enriched counterparts.

## 4. Discussion

Improving the surface properties of biomaterials is an ongoing requirement for better tissue healing in the proximity of an implanted medical device. However, synthesizing biomaterials possessing precautionary properties (antibacterial/antiviral) on their surfaces has recently imposed itself as a vital necessity. In the presented study, we synthesized two nanolayered metal phosphate materials, namely α-ZrP and α-TiP and succeeded, relying on the hydrothermal synthesis, to obtain them in a crystalline form, as was confirmed by XRD, HRTEM and SAED, with a relatively narrow range of sizes, varying between few hundreds of nanometers to few micrometers, as was revealed using SEM and TEM studies. These pristine metal phosphates were further thermally treated to be enriched with crystalline silver nanoparticles. The HRTEM analysis confirmed not only the homogenous distribution of silver nanoparticles on the surfaces of α-ZrP and α-TiP crystals, but also their narrow size distribution with AgNPs around 5 nm in diameter and standard deviation around 2.5 nm. This can be attributed to the regime of AgNPs nucleation (high-density heterogeneous nucleation on the surfaces of the metal phosphate layers) and their subsequent controlled growth that is dominated by heat transfer (melt crystallization) resulting in the growth of pure silver nanocrystals [40].

In practice, the biological systems are nanosystems that possess large specific surface area compared to larger-scaled systems [41]. Consequently, narrowly size distributed AgNPs with high specific surface areas allow larger number of Ag atoms to be in the direct vicinity of its surroundings available for biochemical and physicochemical interactions [42]. In addition, the narrow size distribution is crucial to obtain favorable clinical outcomes, because of the resultant uniform physical properties that enhance their biofunctionality [41]. Another factor reported to influence the biological activity of AgNPs is their shape, however, it was determined that it is rather the exposed lattice plane with the {111} facets exhibiting the strongest biocidal effects, because they are high-atom density facets [43]. In our analyses for the acquired HRTEM micrographs (Figure 5), we have mainly observed d*_hkl_*-spacings corresponding to (111) with exposed {101} facets on the silver nanoparticles, which are not the high-Ag density facets, but their dominance could imply uniformity in their biocidal action. Therefore, these factors altogether imply efficient bioactivity for the Ag-enriched α-ZrP and α-TiP, thereafter, more efficient antimicrobial activity at a possible site of implantation.

EDX analysis performed in STEM (not shown) and SEM modes (Figure 3) confirmed that the α-TiP received as twice as the silver content that was received by the α-ZrP. This can be explained in two complementary interpretations. Firstly, the average size of α-TiP nanolayered crystals that appeared on the larger scale in the SEM micrographs are much smaller than α-ZrP crystals. This implies that for a certain weight of the material larger surface areas were accessible for the AgNPs on α-TiP nanolayered crystals compared to the surfaces of α-ZrP nanolayered crystals. Secondly, the PXRD data analysis of the Ag-enriched metal phosphates showed that, beside the appearance of d*_hkl_*-spacings corresponding to the interlayer distances in the monoclinic crystalline structures of α-ZrP and α-TiP [21], additional larger d*_hkl_*-spacings are observed. These d*_hkl_*-spacings are 7.81 Å in Ag-enriched α-ZrP, attributed to the formation of Zr(AgPO_4_)_2_ [44]; and two adjacent peaks at 7.74 Å and 8.12 Å in PXRD pattern of the Ag-enriched α-TiP, though the latter is assigned in the theoretical structure of this phase as the reflection (100) that rarely appears in experimental PXRD patterns, unless the structure is highly disordered (Figure 2). These additional peaks could be attributed to the layered Ag-phases on both structures (α-ZrP and α-TiP) that have become intercalated with silver through an ion exchange process of monovalent cations (H^+^ for Ag^+^). Given that these additional peaks were of higher intensity on the PXRD pattern of the Ag-enriched α-TiP compared to that of the Ag-enriched α-ZrP, (the integration of the area under peaks at 2θ ≈ 11.32° and 11.82° for Ag-enriched α-ZrP phase and the peaks at 2θ ≈ 11.42° and 11.78° for Ag-enriched α-TiP phase, respectively, gives 1:4 and 3:2 ratios), one can deduce that the intercalation capacity of the α-TiP could be higher than the α-ZrP. Consequently, this suggests that silver is present in two forms in the enriched structures: AgNPs and silver cations.

The antimicrobial activity assays revealed that the MIC and MBC could be accomplished using Ag-α-ZrP at half the concentration needed to achieve the same effects using Ag-α-TiP, despite the fact that their pristine phases, α-ZrP and α-TiP, did not have any effect at the investigated concentrations. This suggests that α-ZrP and α-TiP have no antimicrobial effect at these concentrations (up to 100 µg·mL^−1^), but does not deny the possibility of the presence of the so-called “combined effect” of drugs to execute certain functionality in either synergism or antagonism [45]. In that sense, our analyses imply that silver combined with zirconium can provide better results for antimicrobial functionalities.

To this end, one could presume that the lower antimicrobial effect of Ag-α-TiP contradicts with its higher content of silver. This can be elucidated as follows. Since our experimental observations confirmed that the AgNPs are evenly distributed on both structures’ nanolayered crystals’ surfaces (Figure 4), it can be deduced from their EDX spectra and XRD analyses that more silver ions were intercalated into the interlayers of the Ag-α-TiP than those were intercalated into the Ag-α-ZrP. In addition, since the applied standard antimicrobial assay was performed using phosphate buffered saline at physiological pH that in turn does not induce the release of the intercalated silver ions, the only functional silver component in the performed assay was that of the AgNPs. Yet, in stressful biological condition with more acidic pH the silver ions release would be facilitated, and, in this case, more efficient antimicrobial activity could be expected, especially from Ag-α-TiP at much lower concentrations.

Although in our assay we got for every sample the same MIC and MBC, this could be attributed to the choice of the concentrations’ intervals. Usually, MIC is achieved at a lower concentration compared to the MBC. Therefore, we expect that the MIC could have been realized for Ag-α-ZrP at concentration <25 µg·mL^−1^ and for Ag- α-TiP at concentration <50 µg·mL^−1^.

Regarding the cytocompatibility, both pristine nanocrystals appear to be non-toxic up to reasonable concentrations (up to 12.5 mg·mL^−1^) with the advantage for α-TiP over α-ZrP. Many studies have reported on the antimicrobial benefits of incorporating silver as nanoparticles on the surface of biomaterials [46,47,48]. However, the relatively rapid release of silver ions into the surrounding biological media limits their merits and may induce cytotoxicity. For this, using metal phosphates as silver reservoirs with their ability to undergo controlled release of loaded ions could be a good solution for this problem. The cell viability assay revealed that using α-TiP as a repository for AgNPs led to better cytocompatibility compared to Ag-α-ZrP, up to concentrations of around 12.5 mg·mL^−1^. Again, this can be attributed to the suggested higher intercalation capacity of α-TiP that may have had also the consequence of more controlled release of silver compared to that in α-ZrP. This implies that Ag-α-TiP can be utilized safely above the required concentrations we determined to cause MBC (50 µg·mL^−1^) without leading to severe cytotoxicity. On the other hand, this range of concentrations of Ag-α-ZrP that is required to effectively induce the desired antimicrobial activity (25 µg·mL^−1^) with a tolerable cytotoxicity (125 µg·mL^−1^), is much narrower and this imposes its restrictions regarding the biosafety of the suggested material. This finding introduces α-TiP as a competing safer alternative for the widely accepted α-ZrP for long-term successful biomedical applications.

## 5. Conclusions

Using the hydrothermal route, we could synthesize two nanolayered metal phosphates: α-ZrP and α-TiP, which were enriched with narrow-sized crystalline spherical silver nanoparticles, averaged around 5 nm using the melt crystallization technique. The structural assessment confirmed the stability and crystallinity of the α-ZrP and α-TiP, before and after the Ag-enrichment and their range of sizes that are in the nano up to the submicron dimensions. Studying their cytocompatibility revealed their non-cytotoxic effect as pristine materials with a little loss of their biocompatibility when enriched with silver. In that respective α-TiP maintains better biocompatibility compared to α-ZrP. On the other hand, the combined effect of zirconium and silver led to better antimicrobial activity compared to that of titanium and silver at physiological conditions. However, considering the higher intercalation capacity of α-TiP, better performance for Ag-α-TiP would be expected in stressful biological conditions. Therefore, this research confirms the merits of α-ZrP for biomedical applications, but also highlights, the potential beneficial usage of α-TiP for applications serving the medical field that require controlled release of loaded drugs. More in vitro and in vivo investigations should be performed to further confirm the feasibility of these phases for biomedical applications.

## Figures and Tables

**Table 1:** The antimicrobial activity of the Ag-enriched nanocrystals (the values indicated, in µg·mL^−1^, are the materials concentrations that induced the MIC or MBC).

Sample	MIC	MBC
Ag-α-ZrP	25	25
Ag-α-TiP	50	50

**Table 2:** The CCK-8 proliferation assay of Saos-2 cultured on different concentrations of samples after incubation for 7 days. The average values shown are for the viability rate calculated as thepercentage ratio of the absorbance at wavelength 480 nm for the samples, to the corresponding absorbance for control cells incubated without samples in the same microplate. The standard deviation varied between 5–17.5 %. Cell death was the main regime in all underlined conditions as per the color observations.

Phase Concentration	α-ZrP	Ag-α-ZrP	α-TiP	Ag-α-TiP
1.25 µg·mL^−1^	87.30	61.18	75.02	86.25
12.5 µg·mL^−1^	83.31	58.55	95.65	100.00
125 µg·mL^−1^	76.55	32.13	100.00	92.97
1.25 mg·mL^−1^	60.99	20.66	85.70	44.66
12.5 mg·mL^−1^	50.81	20.44	73.63	21.64
125 mg·mL^−1^	19.22	19.94	10.81	15.55

## Data Availability

The data presented in this study are available on request from the corresponding author.

## References

[1] Brown E., Wright G. (2016). Antibacterial Drug Discovery in the Resistance Era. Nature.

[2] Bhanja P., Na J., Jing T., Lin J., Wakihara T., Bhaumik A., Yamauchi Y. (2019). Nanoarchitectured Metal Phosphates and Phosphonates: A New Material Horizon toward Emerging Applications. Chem. Mater..

[3] Lin R., Ding Y. (2013). A Review on the Synthesis and Applications of Mesostructured Transition Metal Phosphates. Materials.

[4] Kraus K.A., Phillips H.O. (1956). Adsorption on Inorganic Materials. I. Cation Exchange Properties of Zirconium Phosphate. J. Am. Chem. Soc..

[5] Clearfield A., Stynes J.A. (1964). The Preparation of Crystalline Zirconium Phosphate and Some Observations on Its Ion Exchange Behaviour. J. Inorg. Nucl. Chem..

[6] Bullo S., Hussein M. (2015). Inorganic Nanolayers: Structure, Preparation, and Biomedical Applications. Int. J. Nanomed..

[7] Xiao H., Liu S. (2018). Zirconium Phosphate (ZrP)-based Functional Materials: Synthesis, Properties and Applications. Mater. Des..

[8] Contreras-Ramírez A., Tao S., Day G.S., Bakhmutov V.I., Billinge S.J.L., Zhou H.-C. (2019). Zirconium Phosphate: The Pathway from Turbostratic Disorder to Crystallinity. Inorg. Chem..

[9] Bashir A., Ahad S., Malik L.A., Qureashi A., Manzoor T., Dar G.N., Pandith A.H. (2020). Revisiting the Old and Golden Inorganic Material, Zirconium Phosphate: Synthesis, Intercalation, Surface Functionalization, and Metal Ion Uptake. Ind. Eng. Chem. Res..

[10] Huang H., Li M., Tian Y., Xie Y., Sheng X., Jiang X., Zhang X. (2020). Exfoliation and Functionalization of α-Zirconium Phosphate in one Pot for Waterborne Epoxy Coatings with Enhanced Anticorrosion Performance. Prog. Org. Coat..

[11] Ding H., Khan S.T., Aguirre K.N., Camarda R.S., Gafney J.B., Clearfield A., Sun L. (2020). Exfoliation of α-Zirconium Phosphate Using Tetraalkylammonium Hydroxides. Inorg. Chem..

[12] Baker J., Xia F., Zhu Z., Zhang X., Sue H.-J. (2020). α-Zirconium Phosphate Nanoplatelets with Covalent Modifiers for Exfoliation in Organic Media. Langmuir.

[13] Tang M., Yang T., Zhang Y. (2016). A Brief Review on α-Zirconium Phosphate Intercalation Compounds and Nano-composites. Sci. China Technol. Sci..

[14] Espina A., Trobajo C., Khainakov S.A., García J.R., Bortun A.I. (2001). Intercalation of n-Alkylamines into Layered Materials: A Method for the Recognition of Isomorphism in Semicrystalline Compounds. J. Chem. Soc. Dalton Trans..

[15] Trobajo C., Khainakov S.A., Espina A., García J.R. (2000). On the Synthesis of α-Zirconium Phosphate. Chem. Mater..

[16] Kaschak D.M., Johnson S.A., Hooks D.E., Kim H.-N., Ward M.D., Mallouk T.E. (1998). Chemistry on the Edge: A Microscopic Analysis of the Intercalation, Exfoliation, Edge Functionalization, and Monolayer Surface Tiling Reactions of α-Zirconium Phosphate. J. Am. Chem. Soc..

[17] Alberti G., Cardini-Galli P., Costantino U., Torracca E. (1967). Crystalline Insoluble Salts of Polybasic Metals. I. Ion-exchange Properties of Crystalline Titanium Phosphate. J. Inorg. Nuclear Chem..

[18] Alberti G., Torracca E. (1968). Crystalline Insoluble Salts of Polybasic Metals. II. Synthesis of Crystalline Zirconium or Titanium Phosphate by Direct Precipitation. J. Inorg. Nuclear Chem..

[19] Llavona R., Suárez M., García J.R., Rodríguez J. (1989). Lamellar Inorganic Ion Exchangers. Alkali Metal Ion Exchange on α- and γ-Titanium Phosphate. Inorg Chem..

[20] García-Glez J., Trobajo C., Adawy A., Amghouz Z. (2020). Exfoliation and Europium (III)-functionalization of α-Titanium Phosphate via Propylamine Intercalation: From Multilayer Assemblies to Single Nanosheets. Adsorption.

[21] Albitres G.A.V., Cestari S.P., Freitas D.F.S., Rodrigues D.C., Mendes L.C., Neumann R. (2020). Intercalation of α-Titanium Phosphate with Long-chain Amine Aided by Short-chain Amine. Appl. Nanosci..

[22] Valencia Albitres G.A., Cestari S.P., Malafaia Macedo K.R., Mendes L.C., Cruz M.O., Filho M.F., Araújo A.S. (2019). Poly (ethylene terephthalate)/Titanium Phosphate Nanocomposites: Effect of Fillers on Thermal, Crystallographic Diffraction, Molecular Mobility, and UV-Vis Absorption. J. Thermoplast. Compos. Mater..

[23] García-Glez J., Trobajo C., Khainakov S.A., Amghouz Z. (2017). α-Titanium Phosphate Intercalated with Propylamine: An Alternative Pathway for Efficient Europium (III) Uptake into Layered Tetravalent Metal Phosphates. Arab. J. Chem..

[24] Nakamura J., Endo K., Sugawara-Narutaki A., Ohtsuki C. (2020). Human Stem Cell Response to Layered Zirconium Phosphate. RSC Adv..

[25] Hosseinzadeh R., Khorsandi K. (2019). Photodynamic Effect of Zirconium Phosphate Biocompatible Nano-bilayers Containing Methylene Blue on Cancer and Normal Cells. Sci. Rep..

[26] Kalita H., Kumar B.N.P., Konar S., Tantubay S., Mahto M.K., Mandal M., Pathak A. (2016). Sonochemically Synthesized Biocompatible Zirconium Phosphate Nanoparticles for pH Sensitive Drug Delivery Application. Mater. Sci. Eng. C.

[27] Saxena V., Díaz A., Clearfield A., Batteas J.D., Hussain M.D. (2013). Zirconium Phosphate Nanoplatelets: A Biocompatible Nanomaterial for Drug Delivery to Cancer. Nanoscale.

[28] Díaz A., González M.L., Pérez R.J., David A., Mukherjee A., Báez A., Clearfield A., Colón J.L. (2013). Direct Intercalation of Cisplatin into Zirconium Phosphate Nanoplatelets for Potential Cancer Nanotherapy. Nanoscale.

[29] Korneikov R.I., Aksenova S.V., Ivanenko V.I., Lokshin E.P. (2018). Stability of Titanyl Hydrogen Phosphates in Aqueous Media. Inorg. Mater..

[30] Nocchetti M., Donnadio A., Vischini E., Posati T., Ravaioli S., Arciola C.R., Campoccia D., Vivani R. (2019). Zirconium Carboxyaminophosphonate Nanosheets as Support for Ag Nanoparticles. Materials.

[31] Geng Y., Dalhaimer P., Cai S., Tsai R., Tewari M., Minko T., Discheret D.E. (2007). Shape Effects of Filaments versus Spherical Particles in Flow and Drug Delivery. Nat. Nanotechnol..

[32] Decuzzi P., Ferrari M. (2008). The Receptor-Mediated Endocytosis of Nonspherical Particles. Biophys. J..

[33] Ma Z., Yin H., Overbury S.H., Dai S. (2008). Metal Phosphates as a New Class of Supports for Gold Nanocatalysts. Catal. Lett..

[34] Bellezza F., Cipiciani A., Costantino U., Negozio M.E. (2002). Zirconium Phosphate and Modified Zirconium Phosphates as Supports of Lipase. Preparation of the Composites and Activity of the Supported Enzyme. Langmuir.

[35] Clearfield A. (1984). Group IV Phosphates as Catalysts and Catalyst Supports. J. Mol. Catal..

[36] Roberto Monteiro D., Fernando Gorup L., Satie Takamiya A., Colla Ruvollo-Filho A., Rodrigues de Camargo E., Barros Barbosa D. (2009). The Growing Importance of Materials that Prevent Microbial Adhesion: Antimicrobial Effect of Medical Devices Containing Silver. Int. J. Antimicrob. Agents.

[37] Panácek A., Kvítek L., Prucek R., Kolár M., Vecerová R., Pizúrová N., Sharma V.K., Nevecna T., Zboril R. (2006). Silver Colloid Nanoparticles: Synthesis, Characterization, and their Antibacterial Activity. J. Phys. Chem. B.

[38] Prabhu S., Poulose E.K. (2012). Silver Nanoparticles: Mechanism of Antimicrobial Action, Synthesis, Medical Applications, and Toxicity Effects. Int. Nano Lett..

[39] Wypij M., Jędrzejewski T., Ostrowski M., Trzcińska J., Rai M., Golińska P. (2020). Biogenic Silver Nanoparticles: Assessment of Their Cytotoxicity, Genotoxicity and Study of Capping Proteins. Molecules.

[40] Ulrich J., Bülau H.C. (2002). Melt Crystallization. Handbook of Industrial Crystallization.

[41] Danaei M., Dehghankhold M., Ataei S., Hasanzadeh Davarani F., Javanmard R., Dokhani A., Khorasani S., Mozafari M.R. (2018). Impact of Particle Size and Polydispersity Index on the Clinical Applications of Lipidic Nanocarrier Systems. Pharmaceutics..

[42] Marambio-Jones C., Hoek E.M. (2010). A Review of the Antibacterial Effects of Silver Nanomaterials and Potential Implications for Human Health and the Environment. J. Nanopart. Res..

[43] Pal S., Tak Y.K., Song J.M. (2007). Does the Antibacterial Activity of Silver Nanoparticles Depend on the Shape of the Nanoparticle? A Study of the Gram-Negative Bacterium *Escherichia coli*. Appl. Environ. Microbiol..

[44] Clearfiled A., Cheng S. (1980). On the Mechanism of Ion Exchange in Zirconium Phosphates. XXX. Exchange of Silver Ion on α-Zirconium Phosphate. J. Inorg. Nucl. Chem..

[45] Russ D., Kishony R. (2018). Additivity of Inhibitory Effects in Multidrug Combinations. Nat. Microbiol..

[46] Inoue Y., Uota M., Torikai T., Watari T., Noda I., Hotokebuchi T., Yada M. (2010). Antibacterial Properties of Nanostructured Silver Titanate Thin Films Formed on a Titanium Plate. J. Biomed. Mater. Res. A.

[47] Cabal B., Cafini F., Esteban-Tejeda L., Alou L., Bartolomé J.F., Sevillano D., López-Piriz R., Torrecillas R., Moya J.S. (2012). Inhibitory Effect on in vitro *Streptococcus oralis* Biofilm of a Soda-lime Glass Containing Silver Nanoparticles Coating on Titanium Alloy. PLoS ONE.

[48] Ueno M., Miyamoto H., Tsukamoto M., Eto S., Noda I., Shobuike T., Kobatake T., Sonohata M., Mawatari M. (2016). Silver-Containing Hydroxyapatite Coating Reduces Biofilm Formation by Methicillin-Resistant. BioMed Res. Int..

